# From Avicenna to ASCI: embracing the challenges and opportunities of biomedical research

**DOI:** 10.1172/JCI178180

**Published:** 2024-01-02

**Authors:** Sohail F. Tavazoie

**Affiliations:** Laboratory of Systems Cancer Biology, The Rockefeller University, New York, New York, USA.

Members, guests, and physician-scientists in training, during the past year, I have been incredibly honored to have served as president of ASCI, an organization with a long tradition of supporting physician-scientists in their noble mission to uncover fundamental insights into human biology and pathophysiology and apply these insights toward tomorrow’s cures.

As is customary, I’ll start by telling you about my own journey as a physician-scientist. I was born in Iran, like last year’s president Hossein Ardehali, who must be as relieved as I am to have accomplished the first ever peaceful transition between two Iranian-led governments — so to speak. I’m often asked why there are so many Iranian physicians and scientists. One reason for this, I believe, is our parents’ deep reverence for the tenth century Persian physician-scientist and philosopher Avicenna.

A millennium ago, Avicenna authored the “The Canon of Medicine” (1), a five-volume medical encyclopedia that served as the standard medical textbook in the Middle East and much of medieval Europe until the 17th century (2). In this comprehensive work, he proposed various medical advancements, such as the use of alcohol as an antiseptic, and emphasized the importance of research reproducibility — cautioning against accepting results from single trials of herbal drug experiments. In “On the Science of the Pulse,” Avicenna created a classification system for regular and irregular pulses, linking distinct pulse types with specific physiological or disease states. In “The Book of Healing,” he critiqued past philosophies of scientific inquiry (3), including Aristotle’s *Posterior Analytics* (4), in which universal truths are claimed through induction. Instead, he advocated for scientific experimentation as the purest form of scientific inquiry. Avicenna’s ability to question and build upon past philosophies and norms stemmed from his ability to synthesize insights from a diverse array of disciplines, including mathematics, physics, physiology, astronomy, and alchemy.

All four of my grandparents resided within walking distance of Avicenna’s tomb (Figure 1) in the Iranian city of Hamadan (ancient name Ecbatana). Despite lacking formal education, they each instilled traits in their grandchildren — through nature or nurture — that would ultimately support our endeavors in medicine and research. My maternal grandmother, Mahtab, was the matriarch of our family (Figure 2). Her husband abandoned her soon after childbirth and, because he had not officially divorced her, the law barred her from marriage for seven years. Resourceful and resilient, she taught rug-weaving to local women — a skill she had mastered as a laborer since the age of nine. Together, they crafted over 100 large rugs over the span of many decades. The income earned allowed these women to establish and sustain their families.

Mahtab was a formidable, independent, and compassionate woman who believed her primary duty was to help others. This conviction was best exemplified seven years after her husband had abandoned her, when she helped nurse two gravely ill brothers back to health. When the older brother emerged from his convalescence, he told Mahtab that they were forever indebted to her and asked what they could do to repay her kindness. Without saying a word, Mahtab glanced over at the younger brother — Assadollah — and the rest was history, as they say. My grandfather Assadollah (Figure 2) would become a policeman with a passion for cooking. During his military service, he learned that cooks could avoid battle and became sufficiently proficient that he served for a time as the assistant chef for the king’s sister. Persian cuisine, with its intricate arrays of ingredients and precise preparation methods, requires a level of perseverance that has served me well in the laboratory. Learning the complex recipe for ghormeh sabzi, under my mother’s guidance, was one source of preparation for the challenges and setbacks I would later face in the laboratory.

My parents were the first in their families to attain a formal education. My mother, Shahnaz, excelled academically and aspired to attend university and pursue medical studies in Tehran. Unfortunately, societal norms at the time discouraged women from living away from their families, prompting her mother to disapprove of the move. She thus earned a teaching degree and became a committed teacher. I believe her unfulfilled ambition to attain a medical education strongly influenced her aspirations for her children as well as the risks and sacrifices she would endure to enhance our educational opportunities.

My father studied engineering in Tehran, where the government covered all students’ tuition and living expenses. Specializing in metallurgical engineering, he also acquired knowledge in electrical, chemical, and mechanical engineering. With this foundation, he cofounded a company that produced components for high-voltage electrical transmission lines. My dad’s curiosity and ability to fix anything never ceased to amaze us, inspiring our own pursuits of knowledge.

My three siblings and I had an idyllic early childhood in Tehran (Figure 3), but the revolution and Iran-Iraq war brought about dramatic changes. The aerial bombings of Tehran and other cities; the conscription of young men, including teenagers; and significant social and political upheaval prompted many, including my parents, to seek refuge in other countries. When I was nine, smugglers guided my family across the Pakistan border into the desert. From there, we flew to Spain, and after three months, managed to secure temporary visas to enter the US, where my father eventually obtained a work permit. Following years of uncertainly and anxiety, our family was ultimately granted American residency and citizenship.

My father and mother held professional positions as an engineer and manager, respectively, at a computer disk manufacturing company in Utah. My siblings and I were fortunate to receive excellent public school education in the area. My older brother, Saeed, pursued physics at the University of Utah, where I attended a summer research program for high school students under the mentorship of legendary bacterial geneticist John Roth. Initially seeking lab experience to bolster my medical school application, I soon discovered that science was my true passion. John Roth’s boundless enthusiasm for discovery was incredibly contagious. He would spend an hour each day with us discussing the remarkable molecular events underlying our observations, such as colony growth on an agar plate.

Enthralled by biomedical research, I persuaded my older brother Saeed, who was intent on pursuing graduate studies in physics, to spend an extra year taking biology courses. Despite his initial reluctance, due to a dislike for memorization, he agreed to give it a try. A year later, he embarked on an MD-PhD program in Boston, applying his physics and mathematics expertise to genetics and genomics. As a professor at Princeton and now Columbia University, he founded with others the field of systems biology.

I proceeded to the University of California, Berkeley, where I trained with the pioneering neuroscientist Carla Shatz. She instilled in me the importance of conceptual framing and the value of clear, jargon-free communication. After college, I spent a summer in Doju Yoshikami’s lab in Utah, learning neurophysiology and pharmacology. Doju was an exceptionally generous mentor who emphasized the importance of scientific rigor and even permitted me to bring my 15-year-old brother Masoud to the lab. This early exposure to the lab environment sparked Masoud’s interest in science, leading him to MD-PhD training, residency, and, ultimately, his leadership in the development of new experimental cancer therapeutics in the biotech sector. My younger sister Sima also pursued biology, completing graduate studies and becoming a pediatric physical therapist. A talented athlete, Sima had the honor of joining the US national cycling team (Figure 3).

Following in my older brother’s footsteps, I went to Boston for my MD-PhD training and conducted graduate work with R. Clay Reid on the development of neural connections in the visual system. Clay consistently emphasized the importance of delving deeply into significant problems. Upon completing my graduate work, I faced uncertainty about whether to pursue residency training or postdoctoral studies. After I enjoyed an internal medicine elective, Joel Katz, an inspiring medical residency program director at Brigham and Women’s Hospital persuaded me to give internal medicine residency a chance. He assured me that, if I became dissatisfied, I could leave for a postdoc position at the end of my internship. Heeding his advice, I found medicine residency to be among the most rewarding experiences of my life, ultimately altering my career trajectory. Throughout my clinical years, I became increasingly drawn to cancer and oncology. A dear medical school classmate and my favorite cousin in Iran both passed away from cancer, while my mother was diagnosed with her second cancer. My internship further exposed me to the immense clinical burden of metastatic disease and the lack of therapies that directly targeted it.

Consequently, I joined Bernardo Sabatini’s lab for postdoctoral training, where I learned from him the importance of developing new methods and technologies and began my transition from neuroscience to cancer by studying the role of tumor suppressors in the brain. Following my residency, I pursued a medical oncology fellowship at Memorial Sloan Kettering Cancer Center and subsequently began my independent career at The Rockefeller University. I had previously assumed that Rockefeller mainly focused on basic research, but I was pleasantly surprised by the renowned scientists I met during my interview — including Paul Nurse, Mike Young, Jeff Friedman, and Cori Bargmann — who demonstrated a genuine appreciation for disease research. I later discovered that The Rockefeller University was founded by physician-scientists, including Simon Flexner and Samuel Meltzer, the founding president of ASCI (Figure 4). Remaining true to its roots, today’s Rockefeller is helmed by a quintessential physician-scientist and advocate for our cause, Rick Lifton, who will be receiving the Association of American Physician’s Kober Medal tomorrow for his pioneering work on the genetic and biochemical basis of hypertension.

What insights can our young trainees glean from my experiences? First, my family and I, like many others, encountered challenges that ultimately led to remarkable opportunities. Progressive social programs in Iran granted my parents significantly more opportunities than their own parents had. Similarly, grants, loans, scholarships, work-study programs, and Medical Scientist Training Program funding afforded my siblings and me opportunities to become biomedical researchers and healthcare providers. Second, early exposure to science can pay tremendous dividends by igniting a lifelong passion for the field in young people. Thus, we should all strive to introduce young, impressionable students to the marvels of science, be it through teaching at local schools, conducting interactive lab demonstrations, or, most importantly, mentoring high school and college students in summer programs. It is also important that we expose trainees from disadvantaged and underrepresented backgrounds to science, as they have significantly reduced access to these opportunities than the rest of us. Third, one’s career trajectory need not be set in stone. A meandering path and multidisciplinary training can offer a new perspective on a challenging problem. Finally, the American biomedical research enterprise acts as a potent catalyst for upward mobility, enabling my siblings and me to turn our childhood curiosities into lifelong careers. We must all play our part to ensure the vitality of this enterprise for generations to come.

A critical centerpiece of our biomedical enterprise is the physician-scientist. So what challenges do physician-scientists face? Over decades, past ASCI presidents have eloquently discussed the numerous challenges that have threatened the physician-scientist model. To better understand today’s challenges, we solicited survey responses from our membership, receiving 588 responses. When asked about the most significant problems faced by current physician-scientists, 80% reported funding limitations, while 56% cited administrative burdens. Burnout, length of training, and clinical care overload were identified as the next most significant issues, with each being selected by approximately 40% of respondents. Notably, despite these challenges, 90% of respondents reported a high or very high level of career satisfaction. Concerning funding limitations, many respondents advocated for increased NIH funding to physician-scientists, particularly during the transition to independence and junior faculty stages. They also called for enhanced institutional startup and hard-money support for new physician-scientist faculty. Respondents believed that the ASCI could help by providing mentorship advice and workshops for fellows and junior investigators. Additionally, they felt that improved networking among ASCI members across disciplines, within disciplines, and across generations could provide a much-needed support structure for our community.

So, what new initiatives is the ASCI undertaking to address these formidable challenges? To enhance our Society’s networking and mentorship capacity, I initiated a major overhaul of our website. A key feature of the revamped site is the membership directory, where trainees, junior faculty, and ASCI members can view the entire membership and search for members with specific scientific or clinical expertise, such as biochemistry, genetics, cell biology, oncology, or infectious disease. For example, a trainee considering a fellowship in San Francisco could search for all ASCI members in the area, select their names, read their updated biographies, and view their selected publications. Importantly, we are asking all members to provide a brief video upload, in which they describe their career trajectories, what motivates them and brings them joy as physician-scientists, and, crucially, their scientific area of focus. The new directory will increase the visibility of our membership, enable members to form disciplinary or interdisciplinary affinity groups, help trainees identify labs, mentors, and institutions supportive of physician-scientists, and assist us in advocating for increased funding support for physician-scientists from the NIH. This comprehensive directory is a valuable resource that will showcase the remarkable accomplishments of over 3,400 leaders in biomedical research, bolstering our ability to organize and collectively advocate in support of our mission. I extend my gratitude to John Hawley and Karen Guth (Figure 5) for their tremendous support and management of this initiative, and I strongly encourage all of you to help us by updating your biographies, submitting your select publications and uploading your brief videos soon.

To provide targeted advice for different career stages, we have initiated the ASCI Presidents’ question-and-answer sessions, a webinar series starting this June, in which current and past ASCI presidents will address any questions posed by trainees and physician-scientist faculty on a variety of topics, including grant writing, study sections, faculty chalk talks, mentorship, teaching, startup negotiations, administrative burden, leadership, and the inner workings of the ASCI. We will be reaching back decades to past presidents whose timeless insights will greatly benefit our society.

To support trainees at their most vulnerable stage, we will annually pair pre-faculty physician-scientists — the ASCI Emerging Generation Awardees — and early faculty physician-scientists — the ASCI Young Physician-Scientist Awardees — with newly elected ASCI members who have overlapping scientific interests. Each ASCI member will serve as a long-term career mentor or Big Sib for the junior trainee, helping them navigate the most precarious phase of the physician-scientist career path. Vice President Anna Greka has played a major role in guiding young trainee mentorship, along with Christopher Williams, and I’m thrilled that we will have her continued leadership in this initiative.

To highlight exceptional midcareer physician-scientists who can serve as inspirational role models for our future leaders, I am thrilled to announce our inaugural midcareer physician-scientist awards — an initiative created under Hossein Ardehali and Lorraine Ware’s leadership. Wendy Garrett is the recipient of the Marian Ropes award, while Duane Mitchell is the recipient of the Louise Sullivan award. Wendy and Duane will present their exciting scientific work at next year’s annual meeting. Please join me in congratulating them on their achievements.

Additionally, Ben Humphreys, Anna Greka, and I are at the early stages of organizing a working group that will focus on identifying potential solutions to concerns surrounding grant funding and institutional support for junior physician-scientist faculty. These complex issues demand collective thinking, creativity, advocacy, and action. We eagerly anticipate collaborating with you on these crucial matters.

Having discussed challenges, I will now address the incredible opportunities and tools available to today’s physician-scientist. We can now edit genomes of cells and organisms. Gene therapy for specific disorders is proving effective in humans — as evidenced by the groundbreaking work of our Harrington Prize recipients, Jean Bennett and Al Maguire. The long-standing holy grail protein structure prediction problem has been solved by scientists working at the intersection of machine learning and structural biology, allowing prediction of any natural or synthetic protein from amino acid sequence alone. The UK Biobank database provides access to nearly 500,000 human genomic sequences, with clinical, pathological, metabolomic, proteomic, imaging, and other phenotypic data, enabling disease gene and gene-function discovery at an unprecedented scale.

Cryoelectron microscopy allows us to visualize proteins and complexes that had previously eluded structural determination, while in silico virtual screening of billions of compounds can identify small-molecule ligands that can bind targets with high affinity. Engineered cells can eradicate certain liquid cancers but also have potential to sense and report on incipient disease pathogenesis. Innovative nucleic acid therapeutics, such as the antisense therapy for spinal muscular atrophy and the RNA vaccines for SARS-CoV2, have energized the rare-disease and vaccine fields, respectively. Brain-machine interfaces are enhancing the lives of patients with sensory or motor deficits.

Finally, machine-learning and generative artificial intelligence (AI) are poised to impact all fields and professions. AI will assist us in many administrative tasks in the lab and on the wards. Physician-scientists, armed with AI, will identify previously unappreciated associations between clinical, genomic, and phenotypic information, generating new and exciting hypotheses whose functional testing will lead to new areas of exploration. All of these advances are individually transformative, yet they are available to us simultaneously. No one stands to benefit more from these discovery and therapeutic tools than the physician-scientist, as you are uniquely poised to recognize the most clinically significant problems, have access to the patients, understand the complex molecular and clinical phenotypic space, and can advance novel experimental therapies into the appropriate patient populations.

In response to what the greatest opportunities and rewards of being a physician-scientist are, 93% of our survey respondents chose the option making new discoveries. There have never been more tools available to us to for making such discoveries, and there has never been a more exciting time to be a physician-scientist. So, embrace your inner Avicenna and delve into new disciplines, which are now increasingly accessible to everyone. Evade the paralyzed academic investigator’s disease syndrome (PAIDS), coined by Joe Goldstein (5), and utilize cutting-edge methods to deeply dissect your scientific problem. I also urge you to join me in highlighting the magical, honorable, and indispensable nature of our profession to our trainees. By expressing our enthusiasm for their discoveries, we offer a much-needed counterbalance to the negative opinions that pervade social media.

In closing, I want to express my gratitude for the incredible honor of serving our esteemed Society on Council for these past 6 years. I am grateful to my exceptional Council members. I am delighted to pass the baton to our next president and exemplary leader, Ben Humphreys. As evidenced by our fantastic group of Councilors, and my transition to Past President, our society is in excellent hands.

I would next like to express my profound gratitude to my wife, Isabel Kurth, for her unwavering support; our two children, Siroos and Dario, who keep us constantly engaged; my parents, whose sacrifices made my career possible; and my trainees, whose success is my ultimate reward. Finally, I wish to extend special thanks to two people who are the heart and soul of this exceptional organization: John Hawley, the Executive Director, and Karen Guth, the Managing Director of ASCI (Figure 5). John’s vast knowledge of our organization, its history, and inner workings is truly remarkable. Karen’s adept management of the ASCI’s finances, investments, and sizable staff as well as her organization of our meetings is equally impressive. In my view, Karen and John have contributed more to the physician-scientist cause than anyone else I know, and working with them has been an absolute pleasure. This year marks the 25th anniversary of their leadership service at ASCI. On behalf of the present and past 25 Councils and the entire membership, I extend my heartfelt thanks to Karen and John for their dedication to ASCI. Please join me in conveying our tremendous appreciation for their relentless commitment to this extraordinary organization and our shared mission.

## Figures and Tables

**Figure 1:**
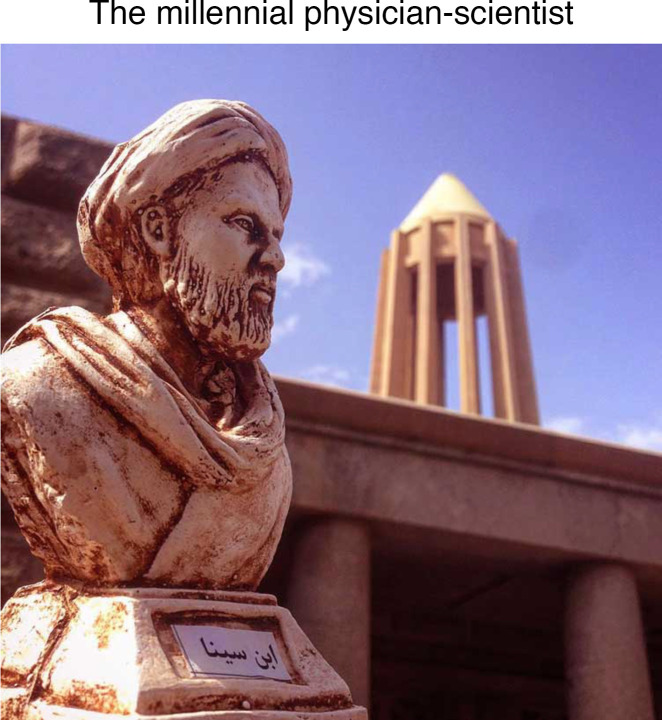
Avicenna’s tomb in Hamadan, Iran.

**Figure 2:**
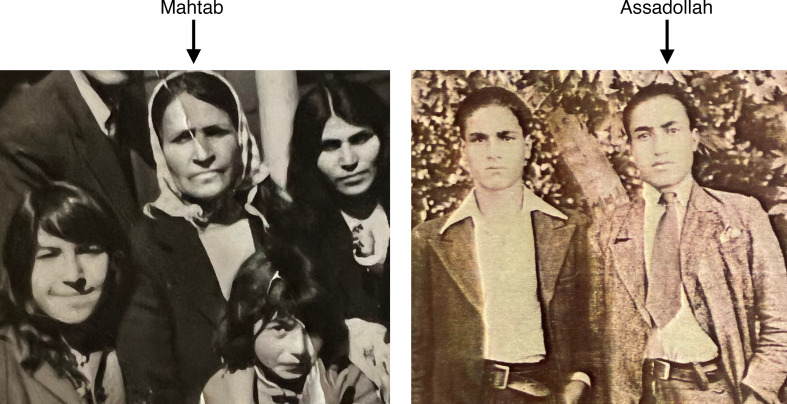
My maternal grandmother, Mahtab, and my grandfather, Assadollah.

**Figure 3:**
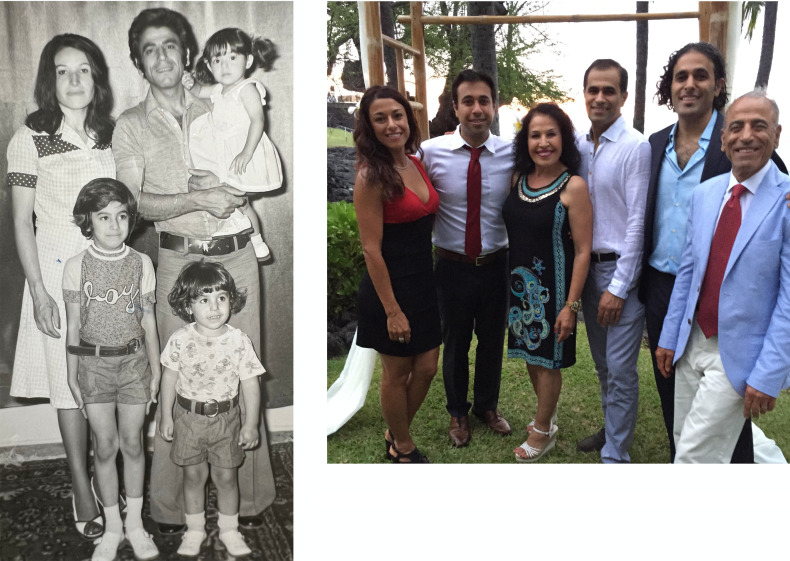
My family.

**Figure 4:**
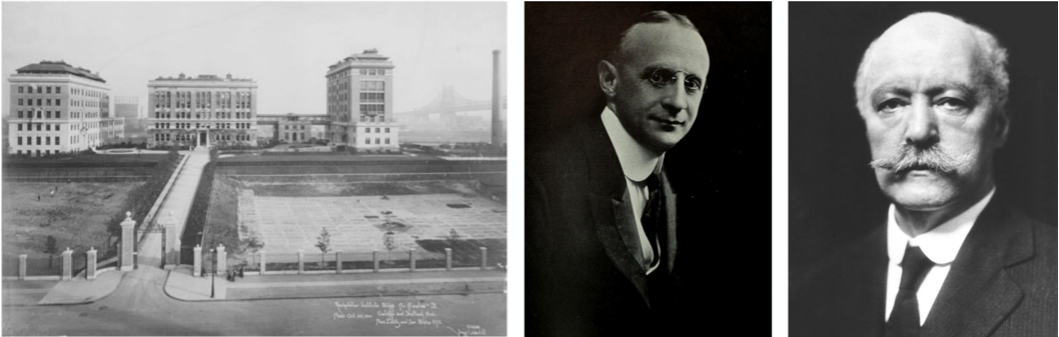
The Rockefeller Institute as well as Simon Flexner and Samuel Meltzer.

**Figure 5:**
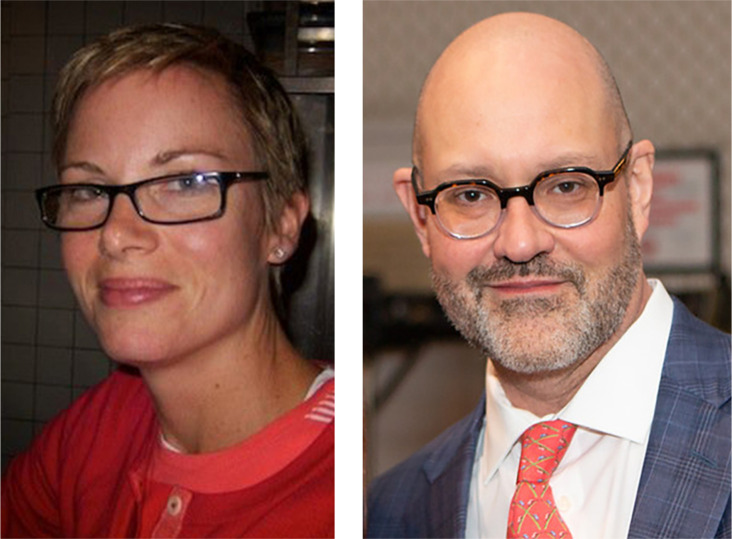
Karen Guth and John Hawley.

## Version 1 01/02/2024

Electronic publication
